# IL17eScan: A Tool for the Identification of Peptides Inducing IL-17 Response

**DOI:** 10.3389/fimmu.2017.01430

**Published:** 2017-10-31

**Authors:** Sudheer Gupta, Parul Mittal, Midhun K. Madhu, Vineet K. Sharma

**Affiliations:** ^1^Metagenomics and Systems Biology Laboratory, Indian Institute of Science Education and Research, Bhopal, Madhya Pradesh, India

**Keywords:** interleukin-17, machine learning, support vector machine, random forest, pro-inflammatory cytokines

## Abstract

IL-17 cytokines are pro-inflammatory cytokines and are crucial in host defense against various microbes. Induction of these cytokines by microbial antigens has been investigated in the case of ischemic brain injury, gingivitis, candidiasis, autoimmune myocarditis, etc. In this study, we have investigated the ability of amino acid sequence of antigens to induce IL-17 response using machine-learning approaches. A total of 338 IL-17-inducing and 984 IL-17 non-inducing peptides were retrieved from Immune Epitope Database. 80% of the data were randomly selected as training dataset and rest 20% as validation dataset. To predict the IL-17-inducing ability of peptides/protein antigens, different sequence-based machine-learning models were developed. The performance of support vector machine (SVM) and random forest (RF) was compared with different parameters to predict IL-17-inducing epitopes (IIEs). The dipeptide composition-based SVM-model displayed an accuracy of 82.4% with Matthews correlation coefficient = 0.62 at polynomial (*t* = 1) kernel on 10-fold cross-validation and outperformed RF. Amino acid residues Leu, Ser, Arg, Asn, and Phe and dipeptides LL, SL, LK, IL, LI, NL, LR, FK, SF, and LE are abundant in IIEs. The present tool helps in the identification of IIEs using machine-learning approaches. The induction of IL-17 plays an important role in several inflammatory diseases, and identification of such epitopes would be of great help to the immunologists. It is freely available at http://metagenomics.iiserb.ac.in/IL17eScan/ and http://metabiosys.iiserb.ac.in/IL17eScan/.

## Background

Human body harbors complex microbial communities which may exist in planktonic forms or as higher order structures termed as biofilms (1). The interaction of the peripheral immune system with these microbes has an essential role in the pathophysiology of different diseases (2). One of the key components of the peripheral immune system is IL-17 family of cytokines, which play regulatory roles in host defense and during inflammatory diseases. They mediate pro-inflammatory responses *via* surface receptors on target cells and play several protective roles in host defense against pathogens at epithelial and mucosal barriers including skin, colon, and lung (3).

The induction of IL-17 by antigens present in gut commensal microbes and its relation with ischemic brain injury/stroke has been well established (2). The intestinal commensal microbes modulate the lymphocyte populations, which lead to various pathological conditions or dysbiosis. Similarly, in case of oral biofilms, the peptides Kgp467–477 of lysine-gingipain protein from *Porphyromonas gingivalis* induce IL-17 and further immunopathology in the case of periodontitis and gingivitis (4). On the other hand, the induction of IL-17 by peptide from agglutinin-like sequence protein in the case of oropharyngeal candidiasis makes it a suitable candidate for immunotherapeutics.

Similarly, there are reports of an increased level of gastric mucosal IL-17 level in response to *Helicobacter pylori* biofilm in mice (5, 6). The pneumococcal surface adhesin A231–268 (PsaA231–268), which is a highly conserved region in clinically relevant *S. pneumonia* strains, can induce an IL-17 response in mice upon infection (7). Furthermore, the Myelin basic protein 85–99 mimicking bacterial peptide can induce IL-17 in humanized transgenic mice (8). Likewise, myocarditogenic mimicry epitopes, such as BAC 25–40 peptide of *Bacillus* sp., induce IL-17 in autoimmune myocarditis in mouse model suggesting a role in its mediation (9). IL-17 secretion can also be triggered when CD4^+^ T-cells encounter viruses. For example, AA242–259 of rotaviral VP6 protein induces an IL-17 response in spleen cells from mice (10). Briefly, the induction of IL-17 in response to various antigens plays a pivotal role in initiation and/or development of several allergic inflammatory responses and autoimmune diseases such as multiple sclerosis (11), autoimmune encephalomyelitis (12), rheumatoid arthritis (13), systemic lupus erythematous (14), Behcet’s disease (15), and psoriasis (16). These evidences suggest that there is a peptide-sequence-specific induction of IL-17 through biofilms and planktonic microbial communities, which further leads to pro-inflammatory responses and pathogenesis. Further the role of selected residues in an epitope was demonstrated by a study carried out by mutating the key binding residues of epitopes and showed that the IL-17-producing CD8^+^ T cells were largely epitope specific (17). Similarly, five key residues essential for T cell activation were identified by replacing the residues with alanine amino acid in env_122–141_ epitope of Friend murine leukemia virus (18).

Several studies have focused on the *in silico* prediction of different types of immune epitopes such as IL4-inducing peptides (19), IFN-gamma inducing major histocompatibility factor (MHC) binders (19), MHC binders (20), T cell epitopes (21, 22), B-cell epitopes (23, 24), and allergenicity (25, 26). However, there are no reports of any study in which the prediction of IL-17 induction by peptides was carried out. In this study, we have developed a classification method to predict the IL-17-inducing property of peptides using sequence-based features from experimentally validated IL-17-inducing and non-inducing epitopes.

## Methods

### Dataset

**Figure 1:**
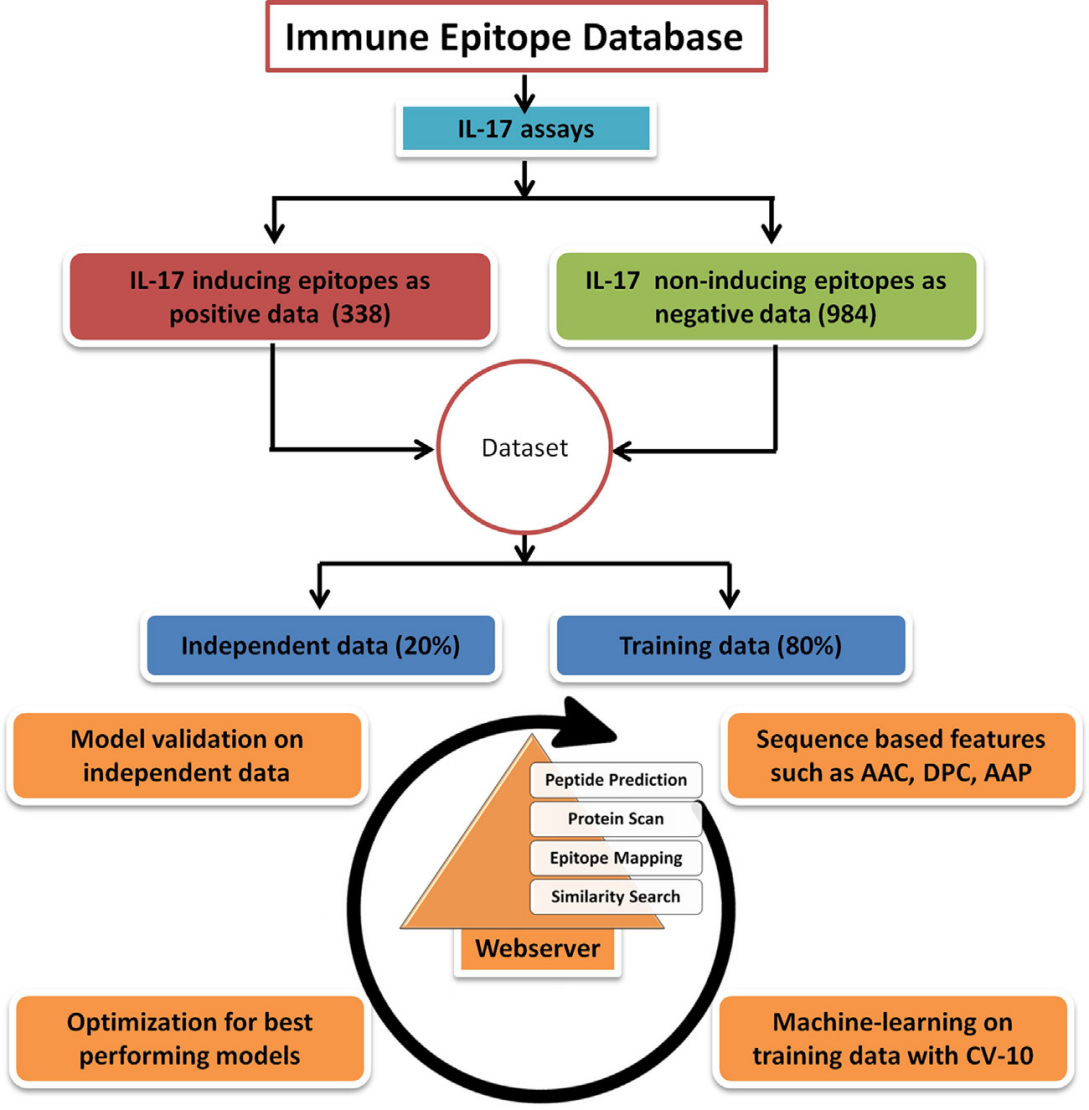
Flowchart showing steps involved in the development of prediction model and web server. The figure shows the steps involved in retrieval of data, training, validation, and construction of the IL17eScan tool.

To ensure a clean and experimentally validated data, the epitope (peptide) sequences reported as IL-17 (IL-17 A or IL-17 F) inducing and non-inducing in different assays were downloaded from the Immune Epitope Database (IEDB) (27). The length of peptides in the epitope data was between 5 and 30 amino acids, and the longer peptides were not included in the study. A total of 338 IL-17-inducing unique epitopes (IIEs) were retrieved and labeled as positive data. The negative data comprised of 984 unique IL-17 non-inducing epitopes (INIEs) which do not elicit an IL-17 response. The peptides in the positive dataset which showed an exact match with the peptides present in the negative dataset were removed from the negative dataset (50 common peptides were removed from 1,034 peptides of negative data). Thus, the sequences of IIEs and INIEs were mutually exclusive with no overlapping peptides in the two groups. Of the total dataset, 80% of the sequences were randomly selected as the training dataset, and 20% data were kept as the validation dataset (Figure 1). The final training dataset contained 271 IIEs (positive data) and 786 INIEs (negative data), whereas the validation dataset consisted of 67 IIEs and 198 INIEs.

To examine the positional amino acid conservation in terminal residues, five residues were cut from both the N′ and C′ terminals of the epitope sequences. The two sample logos (TSLs) were prepared with TSL software (http://www.twosamplelogo.org/) (28).

### Input Features Model Development

#### Composition-Based Features

##### Amino Acid Composition (AAC)

Amino acid composition is the percentage of each amino acid in a peptide of given length. AAC has been widely used in binary classification problems in machine learning (29–31). Each peptide/protein can be represented by percentage composition of the 20 naturally occurring amino acids making a vector size of 20. AAC for each amino acid can be calculated as:

$$\text{AAC}(\text{i})=\frac{\text{Total number of amino acid}(\text{i})}{\text{Total number of all possible amino acids}}\times 100,$$

where AAC(i) is the AAC of the amino acid (i).

##### Dipeptide Composition (DPC)

Dipeptide composition is another widely used input feature for peptide/protein composition-based classification (23, 29, 31), which is calculated using the percentages of the 400 dipeptide combinations. Several immune epitope prediction algorithms have used the DPC-based classification (19, 23). Apart from the composition, DPC additionally provides information about the local arrangements of amino acids in a sequence. Percentage of all possible pairs of amino acids was calculated using the following equation:

$$\text{DPC}(\text{i})=\frac{\text{Total number of dipeptides}(\text{i})}{\text{Total number of all possible dipeptides}}\times 100,$$

where DPC(i) is the dipeptide frequency of dipeptide (i) and the dipeptide (i) is one out of 400 dipeptides.

##### Amino Acid Pair (AAP)

Amino acid pair can be defined as weighted DPC in which each pair carries a weight based on its propensity in the given dataset. The AAP-based feature has been used for the prediction of B-cell epitopes and IL4-inducing epitopes in the past by different authors (19, 23). The AAP feature was calculated as described in the earlier studies (19, 24, 32).

### Machine Learning-Based Prediction Models

#### Support Vector Machine (SVM)

Support vector machine is a supervised machine-learning algorithm that can learn to classify positive and negative data by drawing an optimal hyperplane in high-dimensional feature space separating the two with the highest possible distance. This learning can be used for the classification of unlabeled data. It performs very well on biological data because of its ability to handle large feature spaces and avoid over-fitting, and thus, has been extensively implemented in several immune epitopes prediction tools (19, 33, 34), protein structure prediction (35) and genomic data (36). In this study, SVM^light^ package, available at http://svmlight.joachims.org/ was used for SVM-based predictive modeling. The linear, polynomial, and radial bias function (RBF) kernels were tested using various parameters.

#### Random Forest (RF)

Random forest is an ensemble-based classification and regression method in which a large number of independent decision trees are formed and are then combined to give the final decision. It was implemented in this study as it has a fast and robust algorithm. In this study, the randomForest package in R has been used for developing the classification model. Different mtry and ntrees were tested to build the models.

### Performance Evaluation of Prediction Models

To evaluate and compare the machine-learning methods and prediction models, cross-validation technique was adopted. Cross-validation is a widely accepted method which involves division of the data into two segments. The first part is used to train the model and the other holdout or test data are used to test the model. A 10-fold cross-validation was carried out, where nine parts were used for training of the model, and the 10th one was used for testing the model. The process is iterated 10 times to test all the segments. Results obtained from all the 10 predictions are taken together for measuring the performance using threshold-dependent and threshold-independent parameters. The threshold-independent parameter, area under curve (AUC), was measured using PERF software. ACC, sensitivity (SEN), specificity (SPC), and Matthews correlation coefficient (MCC) were threshold-dependent parameters and were calculated as per the following equations:

$$\text{ACC}=\frac{\text{TP}+\text{TN}}{\text{TP}+\text{FN}+\text{FP}+\text{TN}},$$

$$\text{SEN}=\frac{\text{TP}}{\text{TP}+\text{FN}},$$

$$\text{SPC}=\frac{\text{TN}}{\text{TN}+\text{FP}},$$

$$\text{MCC}=\frac{\left(\text{TP}\times \text{TN})-(\text{FP}\times \text{FN}\right)}{\sqrt{\left(\text{TP}+\text{FP})(\text{TP}+\text{FN})(\text{TN}+\text{FP})(\text{TN}+\text{FN}\right)}},$$

where TP = True Positive, FP = False Positive, FN = False Negative, TN = True Negative.

### Prediction of IL-17-Inducing Peptides in Microbes

To compare the distribution of IL-17-inducing epitopes (IIEs) in different microbes known to induce Th17 responses, or known to induce interleukins other than IL-17 and non-inducing saprophytic microbes (37, 38), the protein sequences of Segmented Filamentous Bacteria, *Staphylococcus aureus, Candida albicans, Listeria monocytogenes, Mycobacterium tuberculosis, Acetobacter aceti* and *Propionibacterium acnes* were retrieved from NCBI. Random synthetic peptides were generated in 10 different sets with 1,000 peptides (15-mers) in each set using in-house Perl scripts and were predicted for their IL-17-inducing property. The IIEs were predicted using the IL17eScan web server.

## Results

### Composition and Position-Based Conservation Analysis

**Figure 2:**
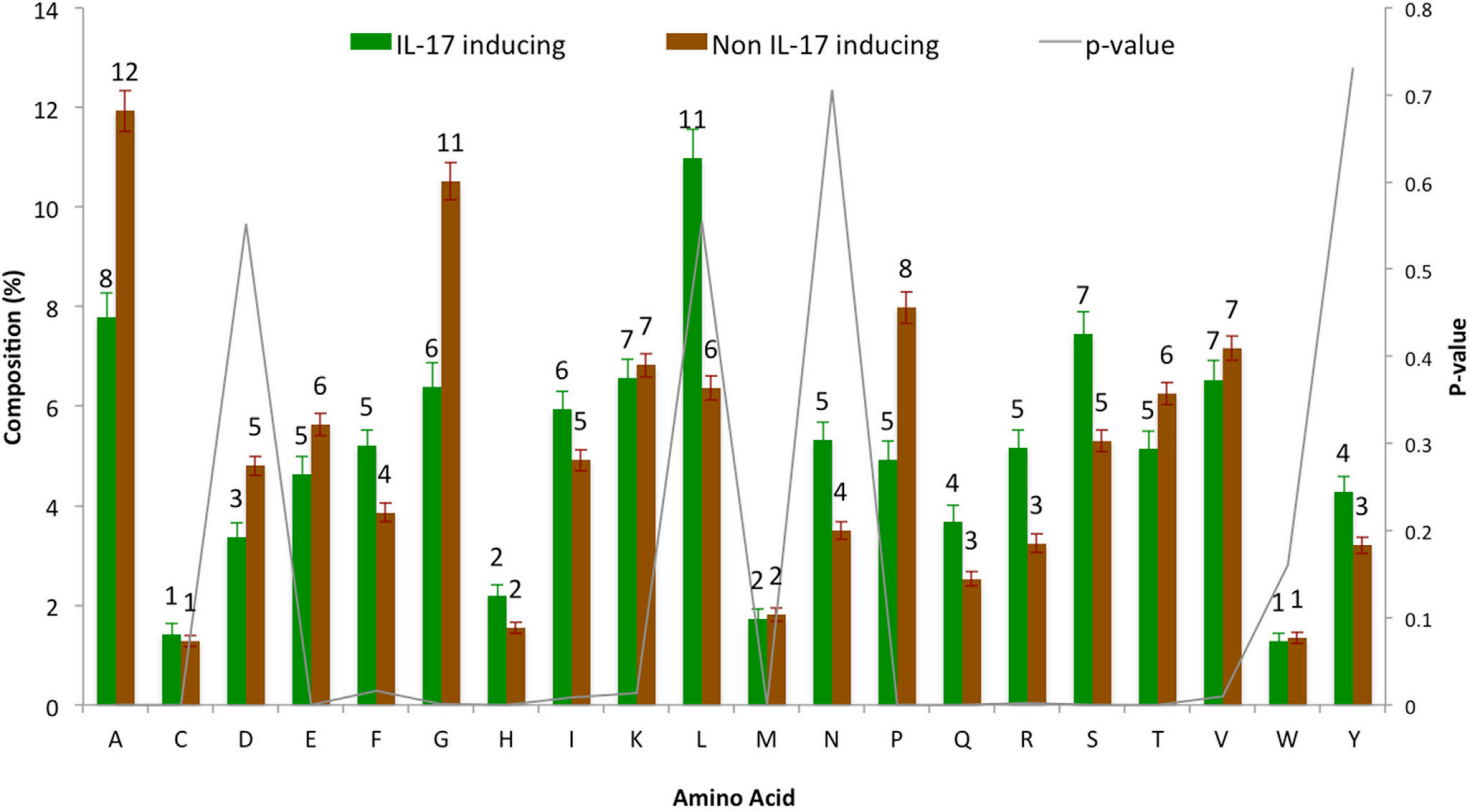
Amino acid composition analysis of IL-17-inducing epitopes (IIEs) and IL-17 non-inducing epitopes (INIEs). The proportions of amino acid in IIEs and INIEs along with the *p*-values (Welch’s *t*-test) are shown.

**Figure 3:**
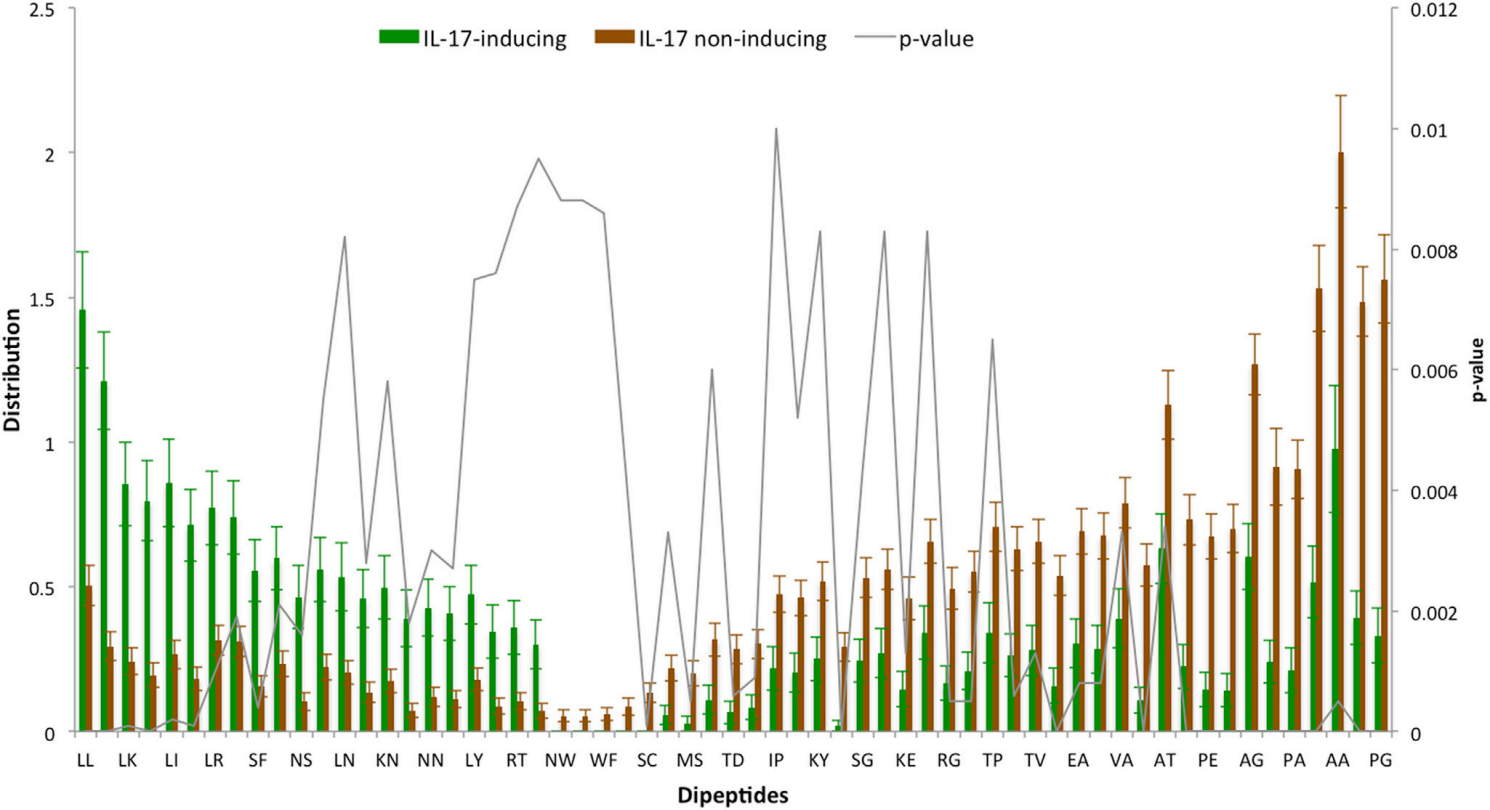
Distribution of dipeptide composition of IL-17-inducing epitopes (IIEs) and IL-17 non-inducing epitopes (INIEs). Significant dipeptides in IIEs and INIEs with *p*-value < 0.01 (Welch’s *t* test) are shown.

The AAC analysis revealed Leu, Ser, Arg, and Asn as the most abundant amino acids in IIEs as compared with INIEs. Similarly, Ala, Asp, Gly, and Pro were found to be rich in INIEs (Figure 2; Data Sheet S1 in Supplementary Material). Furthermore, some dipeptides were found to be significantly abundant (Welch’s *t*-test, *p* < 0.01) in IIEs. The top 10 most abundant and significant dipeptides present in IIEs were LL, SL, LK, IL, LI, NL, LR, FK, SF, and LE, whereas top 10 most abundant and significant dipeptides present in INIEs were PG, GA, AA, GP, PA, PP, AG, GD, PE, and AP (Figure 3; Data Sheet S2 in Supplementary Material).

**Figure 4:**
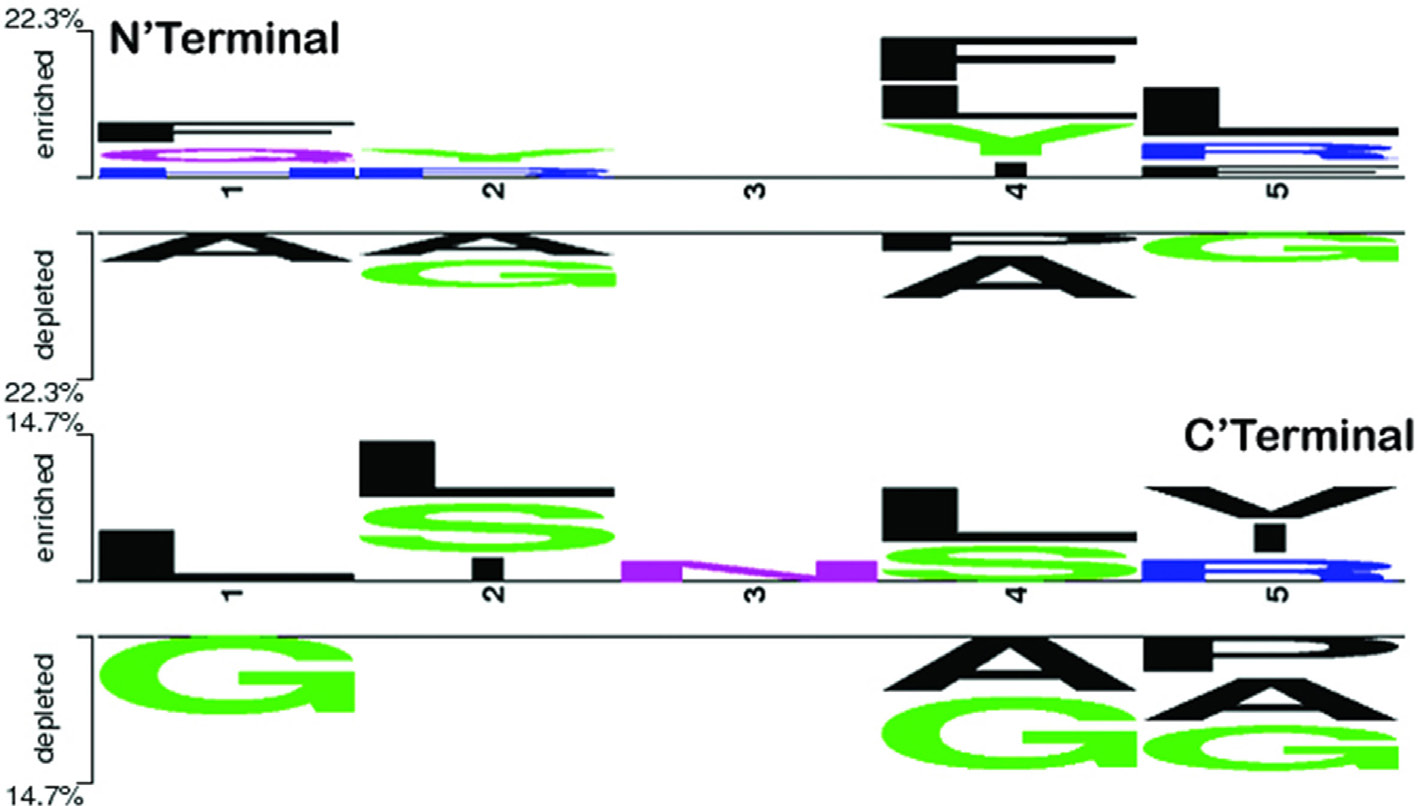
Two sample logo showing positional conservation of five residues at both the terminals (N′- and C′-) in IL-17-inducing epitopes and IL-17 non-inducing epitopes. At both N and C terminals, amino acid L was found abundant and conserved at different positions.

To explore the positional conservation of the amino acid residues, the first five residues from N′- and C′-terminal of epitopes were examined. The TSL analysis revealed the conservation and abundance of Leu residues at various positions (particularly at the N′-terminal), which was also observed as abundant in the compositional analysis of the positive dataset (Figure 4).

### Human Leukocyte Antigen (HLA)-Allele Distribution Analysis

**Figure 5:**
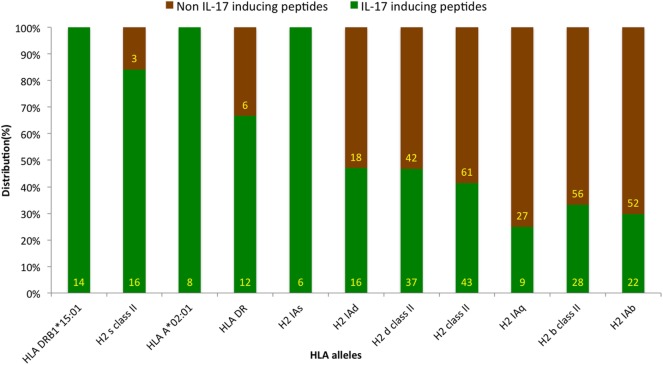
Distribution of human leukocyte antigen (HLA) alleles among assays reporting IL-17-inducing epitopes (IIE) and IL-17 non-inducing epitopes (INIEs). The HLA-allele distribution analysis examines the association of any specific allele with IIEs. The analysis revealed IL-17 induction associated with HLA alleles such as HLADRB1*15:01, H2 s class II, HLAA*02:01, and HLADR. Similarly, HLA alleles, such as H2 Iab, H2 b class II, and H2 Iaq, showed association with INIEs.

Antigenic epitopes are identified by HLA molecules in the host, and the presence of different HLA types is a key determinant of epitope’s action in IL-17 induction (39). The HLA-allele distribution analysis was carried out to examine the association of any specific allele with IIEs. The analysis revealed the association of HLA alleles such as HLADRB1*15:01, H2 s class II, HLAA*02:01, and HLADR with IL-17 induction. Similarly, HLA alleles, such as H2 Iab, H2 b class II, and H2 Iaq, showed association with INIEs (Figure 5; Data Sheet S3 in Supplementary Material). Some previous studies also suggested the association of HLADR alleles with the induction of IL-17, and thus, leading to autoimmune disease such as Rheumatoid arthritis (40).

### Machine Learning-Based Classification

**Figure 6:**
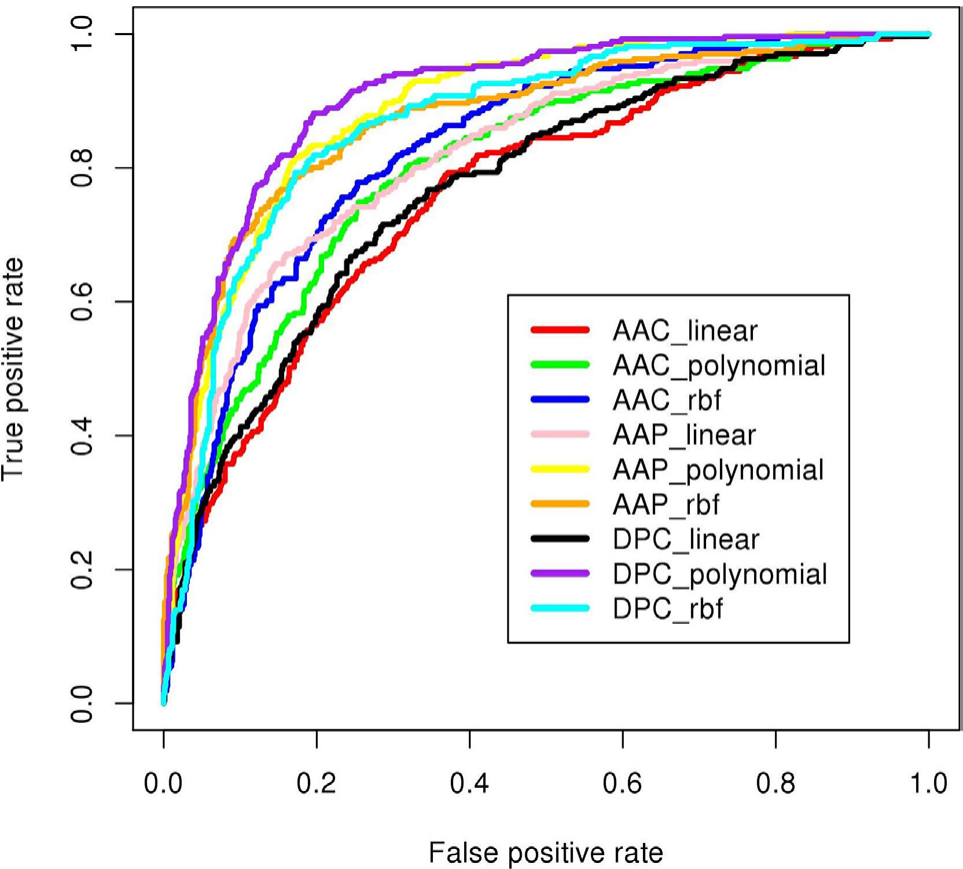
ROC plots of prediction models developed using SVM^light^ as machine-learning technique. The DPC_polynomial model (shown in purple) achieved highest area under curve (AUC = 0.91 shown in Table 1).

The compositional profiles of IIEs and INIEs were found to be different, and thus, could be exploited to classify the epitopes using machine learning-based algorithms. SVM- and RF-based models were developed and evaluated using 10-fold cross-validation. The performance of SVM- and RF-based models on different sequence-based features at various kernels and mtry, respectively are discussed (Tables 1 and 2; Figure 6). Since SVM emerged as the best classification method for IIE and INIE prediction, results of SVM-based models have been mentioned and discussed in the manuscript.

**Table 1:** Performance of support vector machine-based models on different sequence-based features using various kernels.

Feature	Kernel	Thr	Sen	Spec	Acc	Matthews correlation coefficient	Area under curve	Parameters
Amino acid composition	*t*0	−0.9	69.74	69.47	69.54	0.35	0.76	*c*:5
	*t*1	−0.8	71.96	75.7	74.74	0.43	0.8	*d*:3
	*t*2	−0.4	72.69	78.88	77.29	0.47	0.83	*g*:0.005:*c*:1:*j*:5
Dipeptide composition	*t*0	−1	66.79	75.45	73.23	0.39	0.77	*c*:990
	***t*1**	**−0.6**	**87.45**	**80.66**	**82.4**	**0.62**	**0.91**	***d*:2**
	*t*2	−0.6	78.6	82.82	81.74	0.57	0.87	*g*:0.005:*c*:1:*j*:1
Amino acid pair	*t*0	0.1	59.78	88.55	81.17	0.5	0.82	*c*:1
	*t*1	−0.7	78.6	84.1	82.69	0.59	0.89	*d*:2
	*t*2	−0.2	70.11	89.57	84.58	0.6	0.87	*g*:0.01:*c*:5:*j*:1

*Bold fonts signifies the best performance obtained*.

**Table 2:** Performance of random forest-based models on different sequence-based features using various mtry.

Feature	mtry	Acc	Spec	Sens	Matthews correlation coefficient
Amino acid composition	mtry = 8	81.08	83.00	71.01	0.45
	mtry = 7	80.70	82.48	70.81	0.44
	mtry = 4	80.79	82.07	72.97	0.44
Dipeptide composition	mtry = 160	82.12	84.35	71.81	0.49
	mtry = 140	82.12	84.19	72.28	0.49
	mtry = 150	81.65	84.10	70.37	0.48
Amino acid pair	mtry = 45	82.50	83.80	75.60	0.50
	mtry = 35	82.12	83.73	73.84	0.49
	mtry = 25	82.12	83.13	76.28	0.48

#### AAC-Based Models

Support vector machine-based classification using AAC showed the best performance with RBF kernel (*t* = 2), gamma parameter (*g*) = 0.005, trade-off factor (*c*) = 1 and a cost factor (*j*) of 5. This model performed with an accuracy (ACC) of 77.29% and MCC of 0.47 (Table 1). However, MCC at linear and polynomial kernel was found to be 0.35 and 0.43, respectively, which was lesser than the RBF kernel (Table 1; Figure 6).

#### Dipeptide-Based Models

Dipeptide composition was also used as input feature since it harbors more information because of the longer vector length (400). DPC-based models with polynomial kernel (*t* = 1) performed best with parameter *d* = 2. Unlike the AAC-based model which performed best at complex kernel (RBF), the DPC-based model could classify the IIPs from INIEs better with the simpler polynomial kernel. The ACC, MCC, and AUC of the model were found to be 82.4%, 0.62, and 0.91, respectively. Similarly, the models with linear and RBF kernel could only achieve MCC of 0.39 and 0.57, respectively (Tables 1 and 2). The best AUC value of 0.91 was obtained for DPC at polynomial kernel (*t* = 1) (Figure 6).

#### AAP-Based Models

To further improve the performance, weights were given to each dipeptide, and the AAP values were calculated from the DPC as discussed in the Methods section. The model constructed using RBF kernel (*t* = 2) showed the best performance with an ACC of 84.58 and MCC of 0.6. The optimized parameters included gamma parameter (*g*) = 0.01, trade-off factor (*c*) = 5 and a cost factor (*j*) = 1 for this model (Tables 1 and 2; Figure 6).

### Performance on Validation Dataset

After the 10-fold cross-validation, the performance of different SVM- and RF-based models was evaluated on a validation dataset to ensure that there was no over-fitting and the achieved performance of the final model is not due to over-optimization. The performance on the validation dataset are summarized in Table 3 for SVM-based models and Table 4 for RF based models. As mentioned earlier, the best performing models for AAC-, DPC-, and AAP-based features achieved MCC of 0.47 (*t* = 2), 0.62 (*t* = 1), and 0.60 (*t* = 2), respectively, on training data. On the validation dataset, the same models displayed the MCC values of 0.5, 0.57, and 0.52 for AAC, DPC, and AAP, respectively.

**Table 3:** Performance of different support vector machine-based models on validation dataset.

Feature	Kernel	Thr	Sen	Spec	Acc	Matthews correlation coefficient	Area under curve	Parameters
Amino acid composition	*t*0	−0.9	74.63	66.16	68.3	0.36	0.79	*c*:5
	*t*1	−0.8	71.64	70.71	70.94	0.38	0.79	*d*:3
	*t*2	−0.4	80.6	75.76	76.98	0.5	0.86	*g*:0.005:*c*:1:*j*:5
Dipeptide composition	*t*0	−1	62.69	71.21	69.06	0.3	0.76	*c*:990
	***t*1**	**−0.6**	**89.55**	**75.25**	**78.87**	**0.57**	**0.89**	***d*:2**
	*t*2	−0.6	77.61	79.8	79.25	0.52	0.86	*g*:0.005:*c*:1:*j*:1
Amino acid pair	*t*0	0.1	67.16	84.85	80.38	0.5	0.79	*c*:1
	*t*1	−0.7	76.12	79.29	78.49	0.51	0.84	*d*:2
	*t*2	−0.2	67.16	86.36	81.51	0.52	0.84	*g*:0.01:*c*:5:*j*:1

*Bold fonts signifies the best performance obtained*.

**Table 4:** Performance of different random forest-based models on validation dataset.

	mtry	Acc	Spec	Sens	Matthews correlation coefficient
Amino acid composition	mtry = 8	84.15	92.93	58.21	0.56
	mtry = 7	83.77	92.93	56.72	0.54
	mtry = 4	84.53	93.94	56.72	0.56
Dipeptide composition	mtry = 160	83.40	92.42	56.72	0.53
	mtry = 140	83.77	92.93	56.72	0.54
	mtry = 150	83.02	91.92	56.72	0.52
Amino acid pair	mtry = 45	84.91	94.95	55.22	0.57
	mtry = 35	85.28	94.95	56.72	0.58
	mtry = 25	86.42	95.45	59.70	0.62

### IIEs in Biofilm-Forming Bacteria

To examine the epitopes which may modulate host immune system by inducing IL-17 in biofilm-forming microbes in various disease conditions (41), we extracted all the protein sequences of these microorganisms from SwissProt database and analyzed using the prediction pipeline. We identified several IIEs (15-mers) in different proteins belonging to different microorganisms. The top 10 proteins for every microbial species harboring the highest number of epitopes are mentioned in the Data Sheet S4 in Supplementary Material. Among the major predicted IL-17 inducers, “Probable sugar efflux transporter protein” is commonly found in *Haemophilus influenzae* as well as *Klebsiella pneumonia*. Similarly, “Na(+)/H(+) antiporter NhaB protein” from *Proteus mirabilis* and *Pseudomonas aeruginosa* were found to have a large number of IL-17-inducing peptides. DNA polymerase III subunit of *P. mirabilis* involved in urinary catheter cystitis was found to harbor IIE, which corroborates with a previous study on IL-17 induction by DNA polymerase of Human adenovirus 5 (42).

### Prediction of IL-17-Inducing Peptides in Microbes

The IIEs were predicted in microbes known to induce IL-17 response, known to induce other interleukins and in saprophytes using IL17eScan web server. The IIEs were found enriched in the microbes known to induce Th17 responses (Data Sheet S4 in Supplementary Material). *L. monocytogenes* and *M. tuberculosis*, which promote Th1 responses showed a lower representation of IIEs in their proteins (37, 38). A similar lower representation of IIEs was also observed in the case of saprophytic microbes such as *A. aceti* and *P. acnes* (Data Sheet S4 in Supplementary Material). On increasing the threshold to 1, a notable reduction in the percentages of IL-17-inducing proteins was observed, where the percentage was highest (1%) in the case of IL-17-inducing bacteria and the lowest (0.1%) for the bacteria for which there are no reports of their role in IL-17 induction. To further validate the above predictions, random peptides were generated in 10 different sets with 1,000 peptides (15-mers) in each set and were predicted for their IL-17-inducing property at the threshold of 1. Interestingly, none of the synthetic peptides in any of the 10 datasets were predicted to be IL-17 inducing. These results attest the usability of IL17eScan to predict the IIEs in the real datasets.

### Web Server and Tools

A web server “IL17eScan” is constructed to provide the tools for the prediction, virtual screening, and mapping of IIEs. These available modules for prediction incorporate the best performing algorithm (DPC-based model) as default, which runs the queries through a pipeline and classifies the query peptides into IIEs or INIEs. A peptide with a score higher than the threshold is predicted as IL-17 inducing. An increase in the threshold will increase the SPC, and the prediction will become more stringent. As a trade-off between SPC and SEN, an optimal threshold (0.5) is set as default on the web server. However, the user has the flexibility to increase or decrease this threshold and analyze the results as per the requirement. Also, the AAC-based model is provided in all the modules for handling large queries since AAC-based models are faster than DPC-based models due to smaller vector size (20).

#### PepPred

The module “PepPred” classifies one or more proteins/peptide sequence(s) of length ranging from 5 to 30 amino acids into IIEs or INIEs. The stringency of positive prediction can be set using a threshold value provided by the user. Also, the “virtual screening and designing” option has also been provided, which allows the user to select peptides based on their prediction score, modify the query peptides, and resubmit them for prediction. This option carries out substitution of each amino acid of the peptide with other amino acids. After the substitution, for the resubmitted peptides, the results are displayed in the same tabular format with prediction scores. It allows the users to predict the IL-17-inducing nature of the multiple variants of the query peptide, and thus, is useful in assessing the position-specific effects of each amino acid in modulating the IL-17-inducing activity of the peptide.

#### PepScan

In contrast to the “PepPred” module that deals with smaller peptides, the “PepScan” module predicts the antigenic regions in full-length proteins that can potentially induce an IL-17 response in a host. Users are allowed to provide a window length ranging from 5 to 30 peptides which determine the length of peptide sequences considered for prediction. Virtual screening and design option is also available for this module.

#### MetaGScan

To investigate IIEs in amino acid sequence data obtained from metagenomic studies, we have incorporated a separate module “MetaGScan.” This module requires raw translated reads (peptide orfs) from any metagenomic study and identifies the antigenic regions which may induce an IL-17 response. The peptide orf containing the positively predicted epitopes can be aligned for similarity search against the protein sequences present in SwissProt database using BLASTP. As an example, we have included metagenomic reads data from the gut of a diabetes type II patient (processed reads with annotation from https://www.ebi.ac.uk/metagenomics/projects/SRP008047/samples/SRS259434/runs/SRR341581/results/versions/1.0) in this module.

#### EpiScan

To examine the exact occurrence of IIEs on the protein of interest, the EpiScan tool is provided which allows the user to map experimentally validated IIEs from IEDB (27) on the query peptide or proteins. The results are also linked to the related assays available in IEDB.

#### SimSearch

Unlike EpiScan, which searches for exact matches, the “SimSearch” option maps the experimentally validated epitopes to their similar sequences in the query peptide/protein. This module implements Smith–Waterman search algorithm and displays the match along with the links to related assays in IEDB.

## Discussion

Recent advances in metagenomic and high-throughput assay technologies have provided us with new insights into the diversity of human microbiome, and their interaction with host immune system in different inflammatory and autoimmune diseases. Among these interactions, induction of IL-17 is one of the most studied pro-inflammatory responses against pathogens (3, 43, 44). In this study, we have developed an *in silico* method to predict the IL-17-inducing ability of peptides/proteins based on the sequence-based features derived from a set of experimentally validated IIEs (positive set), and non-inducing epitopes (negative set) obtained from the IEDB. Although the IL-17 response can be defined as induction of any cytokine of IL-17 class, the epitope assay data in IEDB were limited only to IL-17 A and IL-17 F cytokines of IL-17 class. Thus, the present tool is aimed only at predicting the IIEs, which is one of the limitations of the tool. Further, the IIEs had lengths ranging from 5 to 30 amino acids except for a few longer epitopes, and thus, the length range of 5–30 amino acids was selected for training and prediction. The non-redundant dataset constructed from the IL-17-inducing and non-inducing peptides ensured no over-fitting or bias due to the presence of multiple instances of the same peptide. The IIEs belonged to 117 unique proteins from 54 different taxa, which further reduced the chances of any bias.

The compositional analysis and positional conservation of residues by TSL revealed that Leu is highly abundant in IIEs as compared with INIEs. The Leu-rich epitopes have also been shown to induce an IL-17 response in different autoimmune diseases such as NLRP3 (autoimmune encephalomyelitis) (45), FLRT2 (systemic lupus erythematosus) (46, 47), and LGI1 (limbic encephalitis) (48–50). A higher abundance of specific residues has been previously observed for epitopes inducing other interleukins and immune cells (17, 18, 21, 22, 51). These findings suggest that a few residues could be associated with IL-17 induction. However, determining the biological significance of these residues in IL-17 induction requires further studies and experimental validations.

The development of IL-17 prediction models was carried out after evaluating multiple machine-learning methods, and the best performing DPC-based SVM classification models with polynomial kernel was incorporated in the web server pipeline for the best results. The DPC-based model performed better than the AAC-based model perhaps due to the larger vector size. However, as a weighted DPC, AAP feature was not able to improve the performance. Given the large vector size and high performance, the models were also scrutinized for over-optimization by testing on a validation dataset. The validation of models on the validation dataset confirmed that the high performance of the models is not due to over-fitting.

Further, the performance of the tool on IL-17-inducing, non-inducing, and saprophytic microbes and on a random peptide set underscores its applicability on real biological datasets and reveals the differences in the percentage of such epitopes in IL-17-inducing and non-inducing organisms. The tool also provides a reliable and reproducible framework for epitope prediction in peptides or proteins from whole genomes and metagenomes. For any prediction-based method, setting an optimal threshold for the selection of hits is one of the limitations, where a lower threshold could result in a higher number of false positives, although it may improve the SEN and *vice versa* for a higher threshold. Thus, we have provided a default threshold to ensure optimal performance; however, the stringency of results should be adjusted by selecting an appropriate threshold by the user.

The availability of experimentally validated IIEs for all classes of IL-17 cytokines will help in further improving the applicability of the tool. The present tool will help in developing a better understanding of the IL-17-inducing property of the peptides and is anticipated to be widely used for the computational identification of IIEs from genomes and metagenomes.

## Conclusion

The propensity of antigens to induce an IL-17 response is of significant importance in the initiation and development of several allergic inflammatory responses and autoimmune diseases. Therefore, the developed machine learning-based tool provides a useful resource for predicting the IL-17-inducing peptides by successfully utilizing the sequence-based signatures of experimentally validated IIEs. To the best of our knowledge, this is the only *in silico* based method available to predict the IIEs in genomic and metagenomic peptides/proteins, and the lead peptides may serve as potential candidates for immunotherapeutics. The IL17eScan is available freely as a web server for academic use.

## Author Contributions

SG developed SVM-based models. PM developed RF-based models. SG, PM, and MM developed web server. SG, PM, MM, and VS analyzed the data and drafted manuscript. SG and VS conceived the work and participated in the design of the study. All the authors read and approved the final manuscript.

## Conflict of Interest Statement

The authors declare that the research was conducted in the absence of any commercial or financial relationships that could be construed as a potential conflict of interest.

## Abbreviations

AAC, amino acid composition; ACC, accuracy; AUC, area under curve; DPC, dipeptide composition; FN, false negative; FP, false positive; IEDB, Immune Epitope Database; IIE, IL-17-inducing epitope; INIE, IL-17 non-inducing epitope; HLA, human leukocyte antigen; MCC, Matthews correlation coefficient; MHC, major histocompatibility factor; RBF, radial bias function; RF, random forest; SEN, sensitivity; SPC, specificity; SVM, support vector machine; TN, true negative; TP, true positive; TSL, two sample logo.
