# Supplementary material for: IL17eScan: A Tool for the Identification of Peptides Inducing IL-17 Response
Source: Front Immunol. 2017 Oct 31;8:1430. doi: 10.3389/fimmu.2017.01430 (PMC5671494; doi:10.3389/fimmu.2017.01430)
Supplement: Supplementary file 4 [file Table_4.docx]

**Supplementary Material 4a: Summary of distribution of IL-17 inducing peptides in microbes (at default threshold)**

| **Species** | **IL-17 Inducing Epitopes** | **IL-17 Inducing Protiens** | **Total Proteins in the Genome** | **% IL-17 Inducing Proteins** |
| --- | --- | --- | --- | --- |
|  |  |  |  |  |
| **Known IL-17 inducing** |  |  |  |  |
| *Candidatus Arthromitus^*^* | 311 | 85 | 1354 | 6.28 |
| *Statphylococcus aureus* | 1149 | 110 | 2574 | 4.27 |
| *Candida albicans* | 1942 | 463 | 6200 | 7.47 |
|  |  |  |  |  |
| **Known to promote other Th response** | |  |  |  |
| *Listeria monocytogenes* | 267 | 81 | 2814 | 2.88 |
| *Mycobacterium tuberculosis* | 104 | 30 | 4088 | 0.73 |
|  |  |  |  |  |
| **Saprophytic Microbes** |  |  |  |  |
| *Acetobacter aceti* | 223 | 73 | 2929 | 2.49 |
| *Propionibacterium acnes* | 105 | 35 | 2366 | 1.48 |
|  |  |  |  |  |

*Segmented Filamentous Bacteria

**Supplementary Material 4b: Summary of distribution of IL-17 inducing peptides in microbes (at threshold >1)**

| **Species** | **IL-17 Inducing Epitopes** | **IL-17 Inducing Protiens** | **Total Proteins in the Genome** | **% IL-17 Inducing Proteins** |
| --- | --- | --- | --- | --- |
|  |  |  |  |  |
| **Known IL-17 inducing** |  |  |  |  |
| *Candidatus Arthromitus^*^* | 42 | 14 | 1354 | 1.03 |
| *Statphylococcus aureus* | 684 | 29 | 2574 | 1.13 |
| *Candida albicans* | 279 | 68 | 6200 | 1.09 |
|  |  |  |  |  |
| **Known to promote other Th response** | |  |  |  |
| *Listeria monocytogenes* | 30 | 13 | 2814 | 0.46 |
| *Mycobacterium tuberculosis* | 17 | 5 | 4088 | 0.12 |
|  |  |  |  |  |
| **Saprophytic Microbes** |  |  |  |  |
| *Acetobacter aceti* | 28 | 9 | 2929 | 0.31 |
| *Propionibacterium acnes* | 11 | 6 | 2366 | 0.25 |
|  |  |  |  |  |

*Segmented Filamentous Bacteria
